# Chitosan/PVA Based Membranes Processed by Gamma Radiation as Scaffolding Materials for Skin Regeneration

**DOI:** 10.3390/membranes11080561

**Published:** 2021-07-26

**Authors:** Maria Helena Casimiro, Andreia Pereira, João P. Leal, Gabriela Rodrigues, Luís M. Ferreira

**Affiliations:** 1 Centro de Ciências e Tecnologias Nucleares (C2TN), Instituto Superior Técnico (IST), Universidade de Lisboa, 2695-066 Bobadela, Portugal; afdi.pereira@campus.fct.unl.pt; 2 Centro de Química Estrutural (CQE), Instituto Superior Técnico (IST), 2695-066 Bobadela, Portugal; jpleal@ctn.tecnico.ulisboa.pt; 3 Departamento de Engenharia e Ciências Nucleares (DECN), Instituto Superior Técnico (IST), Universidade de Lisboa, 2695-066 Bobadela, Portugal; 4 Centro de Ecologia, Evolução e Alterações Ambientais (cE3c), Departamento de Biologia Animal, Faculdade de Ciências da Universidade de Lisboa (FCUL), 1749-016 Lisboa, Portugal; mgrodrigues@fc.ul.pt

**Keywords:** chitosan, PVA, gelatin, biomaterials, gamma irradiation, cell proliferation, skin scaffolds

## Abstract

Some of the current strategies for the development of scaffolding materials capable of inducing tissue regeneration have been based on the use of polymeric biomaterials. Chitosan, in particular, due to its recognized biological activity has been used in a number of biomedical applications. Aiming the development of chitosan-based membranes with improved cell adhesion and growth properties to be used as skin scaffolds allowing functional tissue replacement, different formulations with chitosan of different molecular weight, poly (vinyl alcohol) and gelatin, were evaluated. To meet the goal of getting ready-to-use scaffolds assuring membranes’ required properties and sterilization, preparation methodology included a lyophilization procedure followed by a final gamma irradiation step. Two radiation dose values were tested. Samples were characterized by TGA, FTIR, and SEM techniques. Their hydrophilic properties, in vitro stability, and biocompatibility were also evaluated. Results show that all membranes present a sponge-type inner structure. Chitosan of low molecular weight and the introduction of gelatin are more favorable to cellular growth leading to an improvement on cells’ morphology and cytoskeletal organization, giving a good perspective to the use of these membranes as potential skin scaffolds.

## 1. Introduction

Skin is the largest organ in humans being composed of three primary layers: epidermis, dermis, and hypodermis. In parallel to its protective function against external agents, it has an important role in body thermoregulation and in protein and vitamin D metabolisms since most of the vitamin D produced by the human body comes from the epidermal layer [1,2]. Skin is an extremely complex structure with self-repair capacity acting as a highly efficient sensor regarding temperature, pain, touch, pressure, and light.

Like any other organ, and being the first barrier against external stress, skin is exposed to several aggressions that can induce diseases and severe injuries. Its vital importance makes its replacement essential when, for some reason (burn, accident, disease, etc.), part of the skin is damaged. Thanks to its self-repair ability, a recovery of the skin itself in some cases are expected. However, if the damaged area or the depth of the injured area is large or the problem is due to a disease of the skin itself, treatment may involve a skin graft. It turns out that there is often not enough skin to harvest from the patient (autograft) or that there is a strong possibility that the body will react to the graft harvested from another person (allograft), which in this case, requires immuno-suppressants to ensure that there is no rejection. Xenograft of skin (mainly from pig) can be an alternative procedure [3]. Nevertheless, besides the ethical issues that it can involve, the presence of xenoreactive antibodies in humans results frequently in rapid rejection mechanisms of the grafts. Additionally, in the case of burn patients, immunosuppressive therapy is not advised once they have some degree of immune dysfunction and, consequently, an increased risk of infection [4].

A possible alternative to overcome the above-mentioned difficulties that have been explored in recent years is the use of synthetic skin substitutes that guarantee the protection of the damaged area (helping to prevent infections and promoting skin recovery when this is possible) or even completely replacing the skin [5]. Many of those substitutes are inorganic/organic membranes [6,7,8,9] and a relevant amount of the work involves, in some way, the use of chitin and chitosan (which is a linear polysaccharide formed by N-deacetylation of chitin, composed of N-acetyl glucosamine and glucosamine units linked by β (1–4) glycosidic bonds) [10,11,12,13].

The work developed by the authors under the scope of this subject involves the preparation and functional optimization of 3D biocompatible and biodegradable chitosan-based matrices prepared by gamma irradiation to be used as skin scaffolds designed to promote or replace damaged, missing, or compromised parts of this tissue [11,12]. The scaffolds must thus mime the extracellular matrix (ECM) components and the skin structure [14,15].

The method under development and optimization takes advantage of the recognized biological properties of the natural polymer chitosan (biocompatible, biodegradable, bacteriostatic, and healing) combined with the hydrophilicity and mechanical resistance of synthetic material, in this case, poly (vinyl alcohol) (PVA). The synthesis of the polymeric scaffolds is done by radiation-induced crosslinking. The use of this technology, which does not use harmful initiators or solvents, represents a significant improvement in the current tissue engineering field. By allowing the tailoring of molecular structure, surface roughness, and porosity, ionizing radiation processing is an effective way of preparing, functionalizing, and sterilizing new materials [16,17,18,19,20].

Herein, based on the authors’ previous work [11,12], and aiming to improve cell adhesion and growth properties of the 3D polymeric scaffolds, the optimization, physico-chemical characterization, and in vitro biological evaluation of chitosan/PVA membranes processed by gamma radiation are reported. The study includes the testing of chitosan of different molecular weights (Mw) and the introduction in membranes’ composition of a natural polymer derived from collagen and gelatin, to promote cell adhesion and proliferation [21,22].

## 2. Materials and Methods

### 2.1. Materials

Chitosan of medium molecular weight, M-Chit, (Mw: 190,000–375,000; 75–85% deacetylated chitin), chitosan of low molecular weight, L-Chit, (Mw: 50,000–190,000; ≥75% deacetylated chitin), poly (vinyl alcohol) (Mw: 89,000–98,000; 99+% hydrolyzed), and gelatin from cold-water fish skin (40–50% in H_2_O) were purchased from Sigma-Aldrich (Darmstadt, Germany) and used as received. Acetic acid (min 99.8%, PA) and ethanol (min 99.8%, PA) from Riedel-de Haën (Munich, German), were used as chitosan solvent and neutralizer agent respectively.

### 2.2. Preparation of Chitosan/PVA Based Membranes

Chitosan/PVA-based membranes preparation was based on the authors’ previous work [11,12] which was modified in order to improve membranes’ biological characteristics. In this way, the most promising composition and radiation dose values were selected and used in the present study. Briefly, a 4% (m/V) chitosan solution in 1 % (V/V) aqueous acetic acid was prepared and filtered. A 10% (m/V) PVA solution was also prepared followed by 2 h stirring at 80 °C. At room temperature, specific volumes of both solutions were added to obtain a final solution with 2% in Chit and 5% in PVA of the final volume. To this solution, in some cases, 2 or 4% (m/V) of gelatin from cold-water fish skin was added. These solutions, Chit2/PVA5, Chit2/PVA5/Gel2, or Chit2/PVA5/Gel4 were bubbled with N_2_ gas and a selected volume was transferred to polystyrene Petri dishes before freezing overnight at −26 °C. Thereafter, each Petri dish was neutralized with ethanol, washed with water, and freeze-dried. This freeze-dried procedure comprised of a freezing step at −26 °C for 3 h, followed by another freezing step at −80 °C for 3 h, and finally lyophilization for 48 h. Afterward, small circular pieces of 10 mm diameter were cut, sealed under N_2_ atmosphere, and irradiated at 10 and 15 kGy using a 0.5 kGy·h^1^ dose rate. Irradiations were performed in a Precisa 22 cobalt-60 chamber and routine Amber Perspex dosimeters from Harwell (Oxford, UK) were used for dose monitoring.

### 2.3. Evaluation of Membranes’ Physicochemical Properties

#### 2.3.1. Thermal Stability

The thermal stability of the prepared samples was evaluated by Thermogravimetric Analysis (TGA) using a Q500 thermogravimetric analyzer from TA Instruments (New Castle, PA, USA). The study was carried out under an N_2_ atmosphere in the temperature range between 25 and 500 °C at a heating rate of 10 °C·min^−1^.

#### 2.3.2. Structural Characterization

In order to evaluate the molecular structure and functional groups present in the chitosan/PVA-based membranes, Attenuated Total Reflectance Fourier Transform Infrared Spectroscopy (ATR-FTIR) was used. The equipment was an FTIR spectrophotometer from Thermo Scientific (Nicolet), model iS50, equipped with an ATR module (Waltham, MA, USA), and the spectra were obtained at room temperature between 400–4000 cm^−1^ with a resolution of 4 cm^−1^ and 64 accumulated scans. The spectra of all samples, either non-irradiated and irradiated membranes at 10 and 15 kGy, were obtained in ATR mode.

#### 2.3.3. Morphological Characterization

The surface microstructure of chitosan/PVA-based membranes was analyzed by scanning electron microscopy (SEM). An S-2400 Hitachi microscope (Tokyo, Japan) and 20.0 kV of accelerating voltage were used. All samples were previously Pd/Au coated.

#### 2.3.4. Hydrophilicity

To ascertain the hydrophilicity of the surface of the membranes under study, water contact angles were measured using a goniometer of the brand KSV, model CAM 100. The final value was the mean of 3 measurements (each one resulting from 10 readings obtained in the first 100 ms of contact).

#### 2.3.5. In vitro degradation

The in vitro degradation of the prepared materials was evaluated by immersion in saline solution (NaCl 0.9%) at 36 °C, in order to simulate the saline environment of the human body. The samples were previously weighed (W_0_) and immersed in the solution for 7 days. After that period, the samples were removed and allowed to air dry at room temperature, and the residue was subsequently weighted (W_deg_). The weight loss of the membranes was calculated in terms of percentage of weight loss before and after immersion in saline according to Equation (1):

Weight loss (%) = (W_0_ − W_deg_)/W_0_ × 100 (1)

All measurements were performed in triplicate and the result was expressed as a function of the mean.

### 2.4. In Vitro Evaluation of Membranes’ Biological Properties

Biological assays to evaluate the effect of chitosan/PVA-based matrices on cell adhesion and viability were performed using a Human Caucasian Fetal Foreskin Fibroblast cell line (HFFF2) [12]. The HFFF2 commercial cell line was obtained from the European Collection of Authenticated Cell Cultures (ECACC 86031405, Salisbury, UK). The cells were cultured in Dulbecco’s Modified Eagle Medium (DMEM, Glutamax), supplemented with heat-inactivated fetal bovine serum (FBS) 10 % (*v*/*v*) and streptomycin and penicillin 100 U/mL (all from Gibco, Waltham, USA), and incubated at 37 °C in a humidified atmosphere with 5% of CO_2_. The culture medium was renewed every 2 days and after reaching 80% confluence, cells were trypsinized and resuspended in the culture medium at a concentration of 4 × 10^4^ cell/mL medium.

#### 2.4.1. Cell Viability Assay (almarBlue^®^)

In order to achieve preparation and sterilization in one single step, the membranes used in this study were γ-irradiated in sealed bags. Therefore, irradiated chitosan/PVA- based membranes (ϕ 10 mm) were placed directly in a 48-well tissue culture plate and pre-wetted for 10 min with 200 μL of culture medium to improve samples’ adhesion to the bottom of the well and enable cells migration inside the membranes’ porous structure. After that, a 500 μL suspension of the HFFF2 containing approximately 20,000 cells was seeded on the membranes and cultured for 24 h at 37 °C. Control samples were obtained by growing cells directly on the polystyrene surface of the wells.

Cellular viability was monitored with the alamarBlue^®^ cell viability assay (Life Technologies, Bleiswijk, The Netherlands). On the desired day of culture, the culture medium of each well was replaced by 300 μL of fresh culture medium supplemented with 30 μL of alamarBlue^®^ reagent and incubated for 2 h at 37 °C in a 5% CO_2_ atmosphere. After the incubation period, the solutions were collected and transferred to 96-well plates and the respective optical density (OD) was read at 570 nm with a reference wavelength of 600 nm using a microplate reader (Tecan Spectra, Männedorf, Switzerland). A fresh culture medium was then added to the cells. The background absorbance (blank) was obtained from an “empty scaffold” without cells which were subtracted from the samples’ values. The measurements were made in triplicate and data was expressed as mean ± SD.

#### 2.4.2. Cytochemistry

HFFF2 cells were grown for 24 h in chitosan/PVA-based membranes and glass coverslips (control samples) and then fixed overnight (4 °C) with paraformaldehyde, PFA 4 % (in PBS). They were further permeabilized with 0.2% Triton-X (room temperature, 10 min) and stained at room temperature for 1 h with Methyl Green (1:500 in PBS) and Alexa488 conjugated Phalloidin (1:400 in PBS) (both from Molecular Probes), to assess cell nuclei and actin cytoskeleton, respectively. The samples were mounted in fresh PBS on a glass slide and imaged on a Leica SPE confocal system. Confocal images were analyzed using Image J software. Except for “Control”, which is a single z-slice, images are maximum intensity projections of ~50 μm confocal z-stacks. Basic image manipulation (BandC) was performed for clarity. Fluorescence intensity was not comparable. Green: Phalloidin for actin; Blue: Methyl Green for DNA. Images were pure color RGBs and could be split into multi-channel composite images in FIJI.

## 3. Results and Discussion

### 3.1. Thermal Stability

The weight change measured as a function of temperature was associated with alterations undergone by the sample that resulted from the rupture and/or formation of physical and/or chemical bonds. These changes were usually related to phase transitions, changes in physical state, decomposition or alteration of the molecular structure, loss of water, or chemical alteration of materials [23]. In this way, it was possible to evaluate materials’ structural properties through TGA experiments.

**Figure 1:**
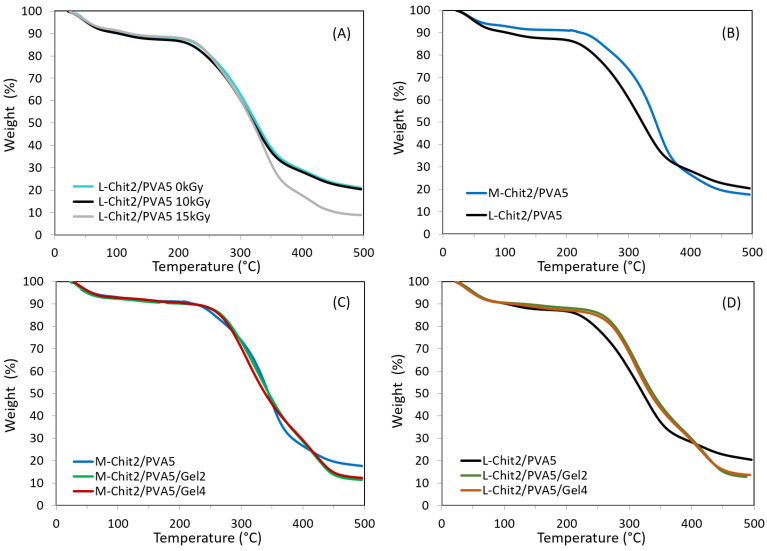
Membranes’ thermogravimetric curves of: (**A**) L-Chit/PVA irradiated at different radiation doses; and irradiated at 10 kGy: (**B**) medium and low Mw chitosan/PVA, (**C**) M-Chit/PVA with different content in gelatin, (**D**) L-Chitosan/PVA membranes with different content in gelatin.

The TGA thermograms of the chitosan/PVA-based membranes in the study are shown in Figure 1.

As can be seen in Figure 1A, the TGA curves’ profile of L-Chit/PVA membranes was remarkably similar among non-irradiated and irradiated membranes within the dose range studied. However, membranes irradiated at 15 kG presented a more pronounced degradation from 350 °C upwards. These results, in terms of the impact of the radiation dose on the membranes’ thermogravimetric behavior, were similar regardless of the type of chitosan used (low or medium molecular weight, L-Chit or M-Chit respectively). However, when comparing chitosan/PVA membranes of different molecular weights with each other, it could be seen that the M-Chit-based membranes presented greater structural stability than the L-Chit ones, even when irradiated as shown in Figure 1B. The observed behavior was most probably due to a longer polysaccharide chain as a consequence of the higher chitosan molecular weight.

Overall, the curves showed a decrease in weight between 5–10% in all membranes up to 100 °C. This loss was usually associated with the loss of water absorbed by the samples. They also showed a second, more pronounced, weight loss, that started at about 225–250 °C for all irradiated and non-irradiated membranes. This variation was associated with the degradation of the saccharide structure, including the decomposition of the deacetylated (and acetylated) units of chitosan [11]. However, it was noticeable that from 350 °C, membranes irradiated at 15 kGy suffered a more accentuated weight loss than the non-irradiated and 10 kGy irradiated ones, suggesting the occurrence of some degradation for higher doses. Thus, this minor stability observed at higher temperatures was probably due to the occurrence of a bigger extension of chain scission at 15 kGy than the one that occurred at 10 kGy.

Concerning the introduction of gelatin into the chitosan-based membranes, results from Figure 1C,D show that it did not change significantly membranes’ thermogravimetric behavior. Even so, some minor profile changes observed were more noticeable in the L-Chit/PVA-based membranes, whereupon a slight increase in the initial degradation temperature was observed with the gelatin introduction. Introducing gelatin, a mixture of water-soluble proteins of high average Mw, in the composition of L-Chit/PVA membranes also appeared to contribute to improving saccharide’ structural stability of those membranes.

### 3.2. Structural Characterization

**Figure 2:**
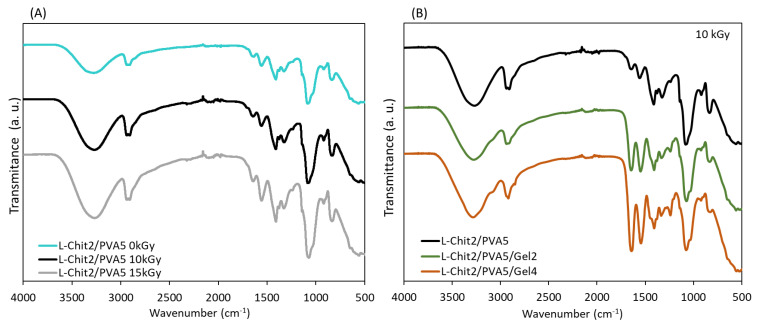
FTIR spectra of low Mw chitosan/PVA membranes: (**A**) non-irradiated and irradiated at 10 and 15 kGy; (**B**) At 10 kGy and different content of gelatin.

Figure 2 presents the FTIR spectra of the chitosan-based membranes in the study. As known, the infrared spectrum of a molecule constitutes its fingerprint which enables useful information to be obtained about its composition and structure.

Figure 2A shows the FTIR spectra of the non-irradiated and γ-irradiated L-Chit/PVA membranes. In both cases, independently of membranes’ composition, it was possible to identify the major FTIR peaks related to the components’ typical pattern.

The literature attributes the peak 3274 cm^−1^ to the vibrational elongation of N-H and O-H of the intermolecular and intramolecular bonds of the hydrogen bonds. Likewise, absorption peaks characteristic of the chitosan were present at 1636 (amide I), 1559 (amide II), and 1328 (amide III) cm^−1^. The absorption peaks at 1142 (antisymmetric elongation of the C-O C bridge) and at 1085 cm^−1^ (C-O elongation of the ring) were also characteristic of the saccharide structure of chitosan. The low-intensity peak at 920 cm^−1^ was characteristic of the presence of the C O-C bridge in the β-glycosidic bonds between the chitosan sugar units. The increase in peak intensity at 1140 and 1556 cm^−1^ showed that the matrix structure was maintained as stable even after irradiation. On the other hand, the peaks at 2939 and 2910 cm^−1^, referring to the C-H elongation of the alkyl groups, and the peak at 1411 cm^−1^, showing elongation of the C-O group, confirmed the presence of PVA in the sample. Furthermore, results evidenced that there was no significant degradation of chitosan in samples irradiated at 15 kGy, since the peak at 1558 cm^−1^ slightly increased in intensity. On the other hand, an increase in peak intensity was also observed at 1410 cm^−1^, suggesting the occurrence of a chemical bond between chitosan and PVA, as well as an increase in the level of crosslinking with the increase in radiation dose [11,12,13,14,15,16,17,18,19,20,21,22,23,24].

Figure 2B shows the FTIR spectra for samples of low Mw chitosan with different content of gelatin at 10 kGy. In this case, besides the same peaks previously mentioned that indicate the presence of chitosan and PVA and the connections between them, the appearance of two characteristic peaks at 1645 and 1544 cm^−1^ due to the presence of gelatin could be observed.

It is important to mention that identical spectra were observed independently of the type of chitosan in use. In this way, and since TGA analysis revealed a better behavior of L-Chit/PVA-based membranes in terms of its structural stability when gelatin was included in the composition, the next set of physicochemical characterization essays were focused on the L-Chit/PVA based membranes.

### 3.3. Morphological Characterization

**Figure 3:**
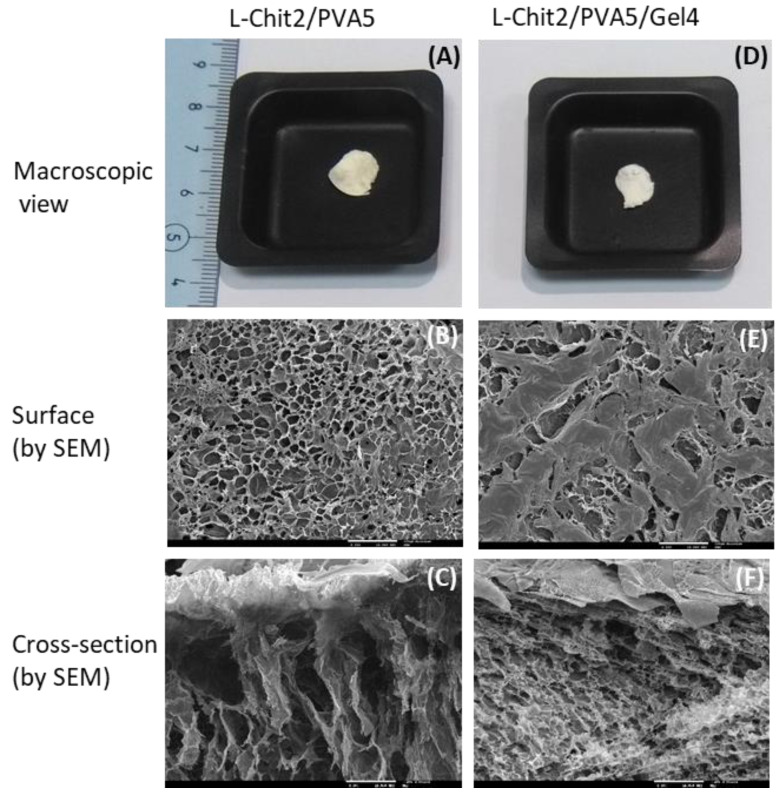
Macroscopic and SEM images of irradiated (10 kGy) L-Chit/PVA-based membranes with and without gelatin. (scale bar 200 µm).

SEM image analysis was performed to evaluate the effect of gelatin introduction in the spatial roughness of L-Chit/PVA irradiated membranes. The obtained images are depicted in Figure 3.

SEM images revealed that both membranes, i.e., with and without gelatin, present, as expected, a porous surface and a sponge-type inner structure. However, it was also noted that the introduction of gelatin promoted some changes in terms of surface roughness and porosity in what seemed to be a coalescence of the surface porous structure.

### 3.4. Hydrophililicity

**Figure 4:**
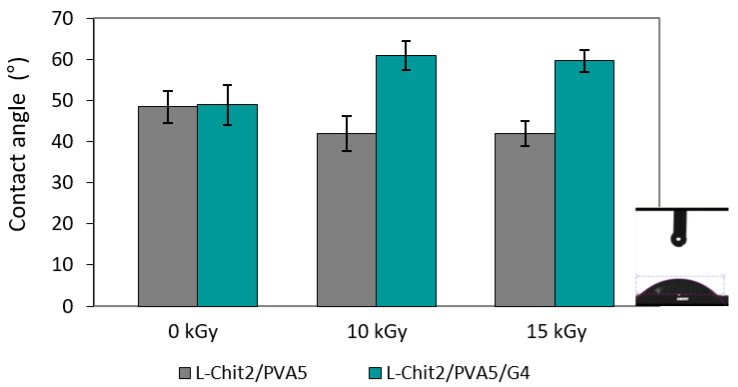
Contact angle of non-irradiated and γ-irradiated low Mw chitosan/PVA-based membranes (n = 3; mean value + SD; the inset on the right side depicts the droplet falling pipe and the water over the surface after contact).

Figure 4 shows the value of the contact angles of the L-Chit/PVA-based membranes.

When a droplet of liquid is deposited on a solid surface, the contact angle, θ, is defined as the angle between the droplet outline and the solid surface. By definition, a liquid wets the solid completely if θ = 0° (hydrophilic material), or partially if 0°< θ < 90° [25].

In the case of the membranes in this study, during the measurements, it was observed that the droplet of water tended to be completely absorbed by the material in approximately 2 s. In addition, as the contact angle of all tested samples was less than 90°, it can be said that all materials have a hydrophilic character. However, it is possible to verify an increase in the contact angle values of Chit/PVA/gelatin membranes when irradiated. As such, it is possible that gelatin is working as a plasticizing agent, conditioning the entry of water into the porous structure of the membranes. In the opposite way, a slight increase in L-Chit/PVA-based membranes hydrophilicity when irradiated (contact angle decreased) was observed, which could be attributed to the incorporation of more OH groups in membranes’ structure due to radiation-induced crosslinking.

### 3.5. In Vitro Degradation

**Figure 5:**
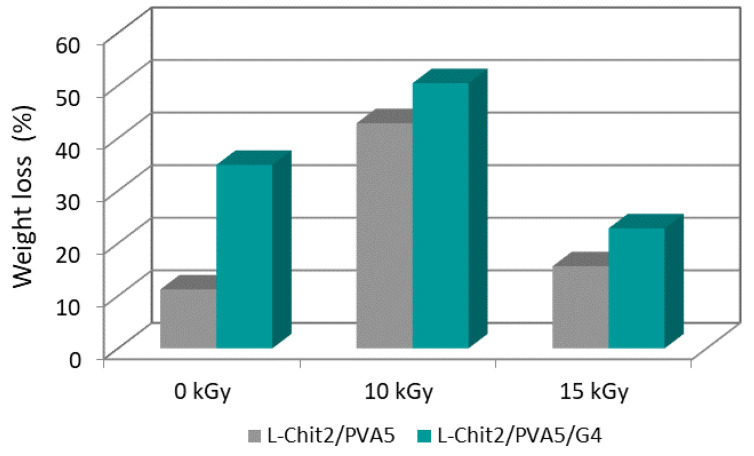
Weight loss of non-irradiated and γ-irradiated low Mw chitosan-based membranes after 7 days immersed in saline solution at 36 °C (error minor than 1% in all cases).

Figure 5 shows the weight loss of the non-irradiated and irradiated L-Chit/PVA-based membranes after 7 days immersed in saline solution at 36 °C.

The membranes irradiated with a γ-radiation dose of 10 kGy lost about 50% of their weight after 7 days immersed in saline solution at 36 °C. This radiation dose seemed to be the one that lead to a higher extension of membranes’ degradation, being the weight loss value higher than the ones of non-irradiated and irradiated at 15 kGy. These results suggest that the radiation dose of 10 kGy introduced changes in the molecular structure that may destabilize its charge balance, leading to higher weight loss when immersed in saline solution. On the other hand, at 15 kGy, the data point that the materials had a more consolidated molecular structure, lead to less of a loss of weight. This trend was observed for the two compositions under study (with and without 4% of gelatin). This fact was corroborated by the higher intensity peaks in FTIR spectra related to membranes irradiated with 15 kGy, which may be associated with a higher degree of crosslinking of the material.

### 3.6. Biological Properties

The biological properties of chitosan/PVA-based membranes were evaluated in vitro through membranes direct contact with fibroblasts. As data from the structural analysis indicated the occurrence of chain scission to a greater extent at 15 kGy, the biological essays were performed using only membranes exposed to 10 kGy.

**Figure 6:**
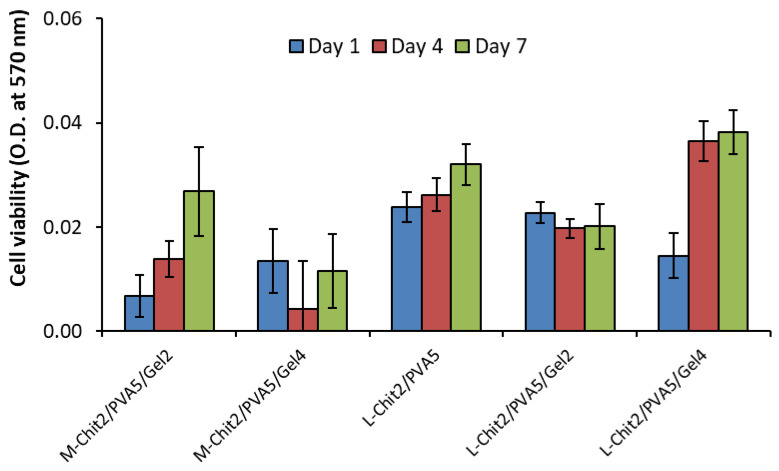
Cellular viability of HFFF2 growing on irradiated (10 kGy) medium and low Mw chitosan-based membranes with and without gelatin, in culture days 1, 4, and 7 (n = 3; mean value ± SD).

Figure 6 shows the results from cells growing on different substrates obtained at days 1, 4, and 7 of culture.

As expected, results indicated that HFFF2 cells attach and are maintained in all irradiated membranes at 10 kGy up to seven days, confirming the non-cytotoxic nature of all the membranes under study. However, data from M-Chit/PVA could not be used due to the detachment of the samples from the bottom of the wells. Nevertheless, results seemed to indicate that L-Chit/PVA-based membranes support the presence of a higher number of fibroblasts and that this effect was improved by the presence of 4% of gelatin. Being a polymer derived from collagen, gelatin contains arginine-glycine-aspartic (RGD) motifs, which is an important sequence in the promotion of cell adhesion [22,23,24,25,26], improving membranes’ biological behavior. On the other hand, cell adhesion also depends on the interaction of the surface charge of the cells with the ones of the substrate. Thus, introducing gelatin in membranes’ composition is likely to promote some destabilization on the positive charge density of chitosan. This effect, depending on the type of chitosan in use and the content in gelatin, can overlap or reduce the potential cell-friendly environment induced by gelatin structure as observed in M-Chit/PVA-based membranes where a 4% in gelatin content displayed a lower number of viable cells than the 2% one.

**Figure 7:**
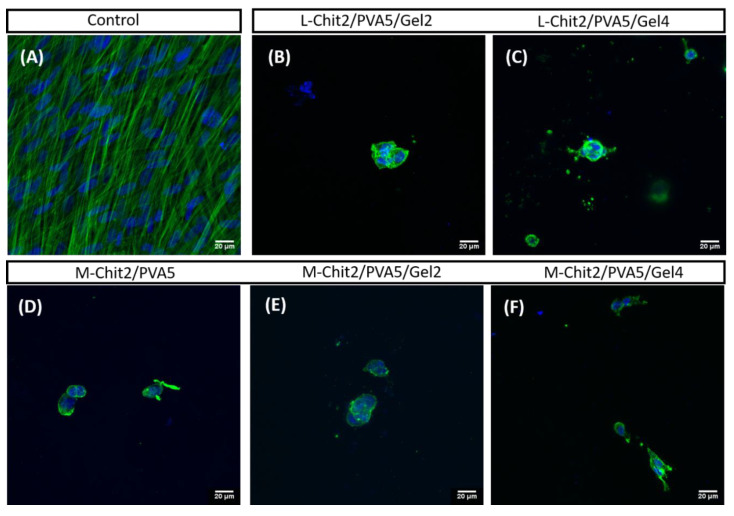
HFFF2 cells growing in culture for 7 days: (**A**) control cells; (**B**–**F**) 10 kGy irradiated Chit/PVA-based membranes (green: actin; blue: DNA). Scale bars: 20 µm.

These observations were in accordance with the data displayed by the confocal images (Figure 7), being that the L-Chit/PVA5/Gel4 membrane displayed a higher number of cells in growth.

Results from the cytochemical stainings performed on day 7 showed that cells growing on irradiated Chit/PVA-based membranes (from (B) to (F)), displayed round morphology and poor actin cytoskeletal organization, as compared to control cells (A), which displayed a fusiform shape and highly oriented bundles of actin microfilaments, similar to what happens in the in vivo fibroblasts. Nevertheless, cells were able to invade the depth of the membrane. Thus, upon comparison, L-Chit/PVA-based membranes with the addition of gelatin were revealed to be more favorable to cellular growth leading to an improvement in cells’ morphology and cytoskeletal organization. These membranes showed suitable characteristics in terms of biocompatibility for the intended use as skin scaffolding materials.

To sum up, it was clear that an increase in irradiation dose within the studied range lead to an increase of membranes’ structural stability and that the major FTIR peaks, related to a typical components pattern, were present before and after irradiation.

Weight loss studies and contact angle measurements of L-Chit/PVA-based membranes showed that the introduction of gelatin leads to higher values in both cases independently of the studied dose (10 and 15 kGy). For the same composition, doses in the study seemed to not induce significant changes in membranes hydrophilicity, however, weight loss at 15 kGy was lower than at 10 kGy suggesting a higher reticulation degree at 15 kGy.

From the biological essays, it was possible to observe that cells adhered to all membranes, which is indicative of the non-cytotoxic nature of the prepared membranes. All the materials and methods used lead to membranes that were biocompatible to the human fibroblasts cell line under study. Results also showed that the use of L-Chit and the introduction of gelatin were favorable to cellular growth leading to an improvement in cells’ morphology and cytoskeletal organization in comparison with the other membranes as evidenced by cytochemistry characterization. Gelatin, having a similar protein composition to collagen, promotes cellular adhesion and proliferation, boosting their natural growth and differentiation [22,23,24,25,26]. The combination of L-Chit with gelatin in membranes composition was a determinant for the improvement of the culture conditions for cells’ growth and organization.

The other undeniable advantage of this method of preparation was the fact that the membranes’ preparation, tailoring, and sterilization could be achieved in one single step (no post-purification processes are required) with the consequent effectiveness and cost reduction of the entire process.

Validation of in vitro results by in vivo regeneration studies to choose the best scaffold (s) will be undertaken in the near future.

## 4. Conclusions

The Chitosan/PVA-based membranes prepared were evaluated in terms of composition, absorbed dose, structural and functional properties, and in vitro biocompatibility (cellular viability, morphology, and cytochemistry).

Through the approach herein presented, it was possible to obtain 3D chitosan-based membranes with a sponge-type inner structure. The synergistic effect of chitosan of low molecular weight (L-Chit) and gelatin in membranes’ characteristics and consequently on their biological behavior, was decisive for the improvement of cell growth and organization. Thus, results show great potential for the application of these membranes as scaffolding materials for skin regeneration. In vivo studies will be used as the next approach to confirm these membranes as potential skin scaffolds.
